# The complete mitogenome of *Calappa japonica* ortmann, 1892 (Decapoda: Calappidae) from the East China Sea

**DOI:** 10.1080/23802359.2026.2657107

**Published:** 2026-04-10

**Authors:** Xing-Xing Zhao, Yi-Feng Fang

**Affiliations:** Zhejiang Museum of Natural History, Hangzhou, China

**Keywords:** Calappidae, box crab, mitochondrial genome, phylogenetic relationships

## Abstract

This study presents the first complete mitochondrial genome of *Calappa japonica* Ortmann, 1892, the fourth genetic resource for the family Calappidae. Its mitogenome (GenBank: PX866701) is 15,773 bp in length and comprises 13 protein-coding genes (PCGs), two rRNA genes, 22 tRNA genes, and one control region (D-loop). Nucleotide composition analysis showed a high AT content (69.3%), with adenine (34.5%) and thymine (34.8%) dominating. Phylogenetic analysis grouped *C. japonica* with *C. granulata* and *C. rissoana* in a monophyletic clade, with *C. bilineata* as the sister to this clade. This work provides mitochondrial genomic resources for future studies on Calappidae systematics and species identification.

## 1. Introduction

Members of the family Calappidae (Decapoda: Heterotremata), commonly known as box crabs, comprise over 88 described species across nine genera, many of which hold economic importance (Bellwood 1996; Zhang and Xu 2023). Among them, *Calappa japonica* Ortmann, 1892 is a widely distributed species inhabiting shallow marine habitats (30–380 m depth) in sandy-mud substrates from Japan and Taiwan (China) to Australia, the Bay of Bengal, East Africa, and the Red Sea (Chen and Sun 2002). Despite its broad distribution, phylogenetic relationships within Calappidae remain poorly resolved, largely due to high morphological similarity among species and considerable ecological variation, leading to persistent taxonomic controversy (Ewers-Saucedo et al. 2016; Camargo et al. 2020).

So far, only three species of Calappidae have complete mitogenomes in GenBank (Lu et al. 2020; Tanduo et al. 2026). Most studies have relied on morphological characters, with genetic data scarce. Therefore, this study aims to characterize the first complete mitogenome of *C. japonica* and investigate its phylogenetic position within the family and its relationships with related groups.

## 2. Materials and methods

### 2.1. Sample collection and preservation

**Figure 1.**
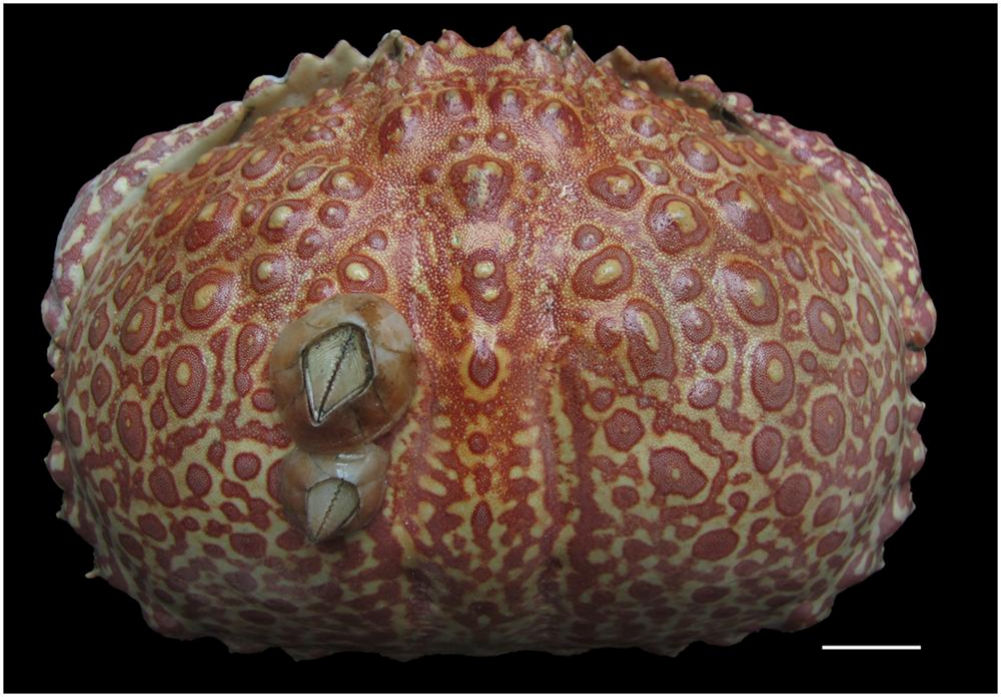
Dorsal view of *calappa japonica* that taken by fei Wu. Scale bar is 2.0 cm.

An individual of *C. japonica* (Figure 1) was collected by trawl net in March 2025 from the southern East China Sea (26°44’N, 123°28’E) at depths of 80–130 m. A pereiopod muscle sample was dissected and immediately preserved in DNA preservation solution (Phygene, Cat# PH1048, Fuzhou, China), which contains Tris, EDTA, and preservative agents. The voucher specimen was deposited in the Zhejiang Museum of Natural History (Xing-Xing Zhao, zhaoxx@zmnh.com), under the voucher number AIACx1077.

### 2.2. DNA extraction, sequencing, and annotation

Genomic DNA was extracted from 20 mg of pereiopod tissue using the CTAB method (Doyle and Doyle 1987). A library was constructed with the MGIEasy^™^ FS DNA Library Prep Kit (MGI, Shenzhen, China) and sequenced on the Illumina NovaSeq 6000 platform (2 × 150 bp), yielding 35,979,550 clean reads. Mapping depth ranged from 105× to 3,178×, with an average coverage of 2,042× (Supplementary Figure S1).

A localized reduction in coverage (105×) was observed within the *ND6* coding region, which exhibits a high AT content (74.3%). This depth is sufficient for reliable assembly and downstream analyses. The mitogenome was assembled using NOVOPlasty v4.3.1 (Dierckxsens et al. 2017) with the *COX1* sequence of *C. bilineata* as a bait and the complete mitogenome of *C. bilineata* (GenBank: MN562587) as a reference. Annotation was performed *via* the MITOS web server (Bernt et al. 2013), and start/stop codons of all protein-coding genes (PCGs) were manually corrected in Geneious (v2025.0.2, Dotmatics, New Zealand). A graphical map of the complete mitogenome was generated using Proksee (Grant et al. 2023).

### 2.3. Phylogenetic analysis

Phylogenetic analysis was conducted in PhyloSuite (Zhang et al. 2025) based on a concatenated matrix of 13 PCGs and two ribosomal RNA (rRNA) genes. A total of 13 complete mitogenomes from species with *COX1* sequence similarity ≥81% to *C. japonica* were included in the phylogenetic analysis. The *Conchoecetes atlas* and *Dynomene pilumnoides* (section Dromiacea) were used as the outgroup (Table 1). Sequences were aligned with MAFFT v7.463 (Katoh and Standley 2013). The alignments were processed separately: PCGs with Gblocks v0.91b (Talavera and Castresana 2007) and rRNA genes with trimAI v1.2 (Capella-Gutiérrez et al. 2009). The trimmed alignments were concatenated into a supermatrix.

**Table 1.** GenBank Accession numbers of sequences included in this study.

Species	Genbank	Length(bp)	References
*Conchoecetes atlas*	PV916619	15955	unpublished
*Calappa bilineata*	MN562587	15606	Lu et al. (2020)
*Calappa granulata*	PV567534	15795	Tanduo et al. (2026)
*Calappa japonica*	PX866701	15773	This study
*Calappa rissoana*	PV582136	15807	Tanduo et al. (2026)
*Carpilius maculatus*	MN381805	15761	Liu et al. (2019)
*Dynomene pilumnoides*	KT182070	16475	Shi et al. (2016)
*Chaceon granulatus*	PQ300092	16135	Kong et al. (2025)
*Charybdis granulata*	MW446891	15774	unpublished
*Charybdis hellerii*	PX672818	15799	unpublished
*Charybdis hongkongensis*	PX672819	15690	unpublished
*Portunus pelagicus*	KT382858	16155	Ma et al. (2016)
*Thalamita danae*	MW309525	15809	unpublished
*Thalamita prymna*	MW309527	15807	unpublished
*Thalamita sima*	MG840650	15831	Zhong et al. (2018)
*Thalamita spinicarpa*	MW309529	15783	unpublished

The optimal partitioning scheme and best-fit substitution models for each partition were determined using ModelFinder v3.2 (Kalyaanamoorthy et al. 2017) under the AICc criterion. A total of 40 partitions were defined based on gene and codon position (Supplementary Table S1). Maximum Likelihood (ML) analysis was performed with IQ‑TREE v2.2.0 (Minh et al. 2020) using partition-specific models and 50,000 ultrafast bootstrap replicates. Bayesian Inference (BI) analysis was conducted using MrBayes v3.2 (Ronquist et al. 2012) based on the partitioning scheme selected by PartitionFinder 2 (Lanfear et al. 2017) under the AICc criterion, yielding 22 partitions (Supplementary Table S2). Two independent MCMC runs of 2,000,000 generations were performed, with the first 25% of samples discarded as burn‑in. Tree was visualized using iTOL (Letunic and Bork 2024).

## 3. Results

**Figure 2.**
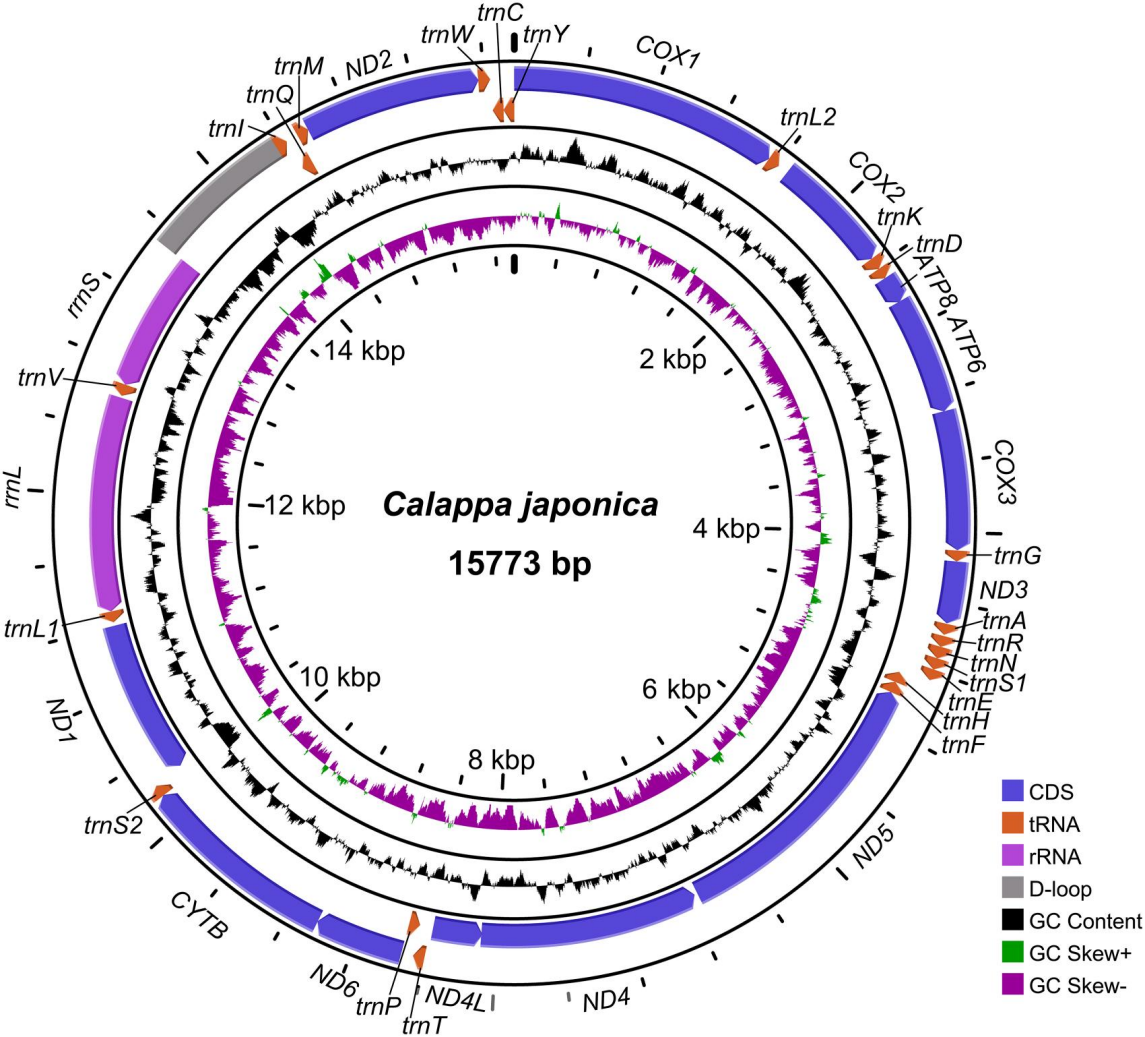
Mitogenome map of *calappa japonica.* Outer (forward strand) and inner (reverse strand) circles display the distribution of PCGs, rRNA genes, and tRNA genes. Genes for proteins and rRNAs are labeled with standard abbreviations. tRNA genes are represented by a single letter for the corresponding amino acid, with two leucine tRNA genes and two serine tRNA genes distinguished by numerals. The map includes annotations for the D-loop region, GC content, and GC skew patterns. GC content and GC skew were calculated using a 50 bp sliding window. The four solid black rings are track dividers.

The assembled mitogenome of *C. japonica* is a circular double-stranded DNA molecule of 15,773 bp (Figure 2), containing 13 PCGs, 22 tRNA genes, two rRNA genes, and a control region (D-loop). Of the 37 genes, 14 (four PCGs, two rRNA genes, and eight tRNA genes) were located on the light strand, whereas the remaining 23 (nine PCGs and fourteen tRNA genes) were transcribed from the heavy strand. The mitogenome showed an AT bias (adenine: 34.5%, thymine: 34.8%, guanine: 10.1%, cytosine: 20.6%).

Initiator codons usage varied: nine PCGs (*COX1*, *COX2*, *COX3*, *ATP8*, *ND2*, *ND4*, *ND4L*, *ND5*, *CYTB*) used ATG, two (*ND3* and *ND6*) used ATT, and two (*ND1* and *ATP6*) utilized ATA. For terminator codons, three PCGs (*COX1 COX2*, *ND5*) terminated with a single T, four (*CYTB*, *ATP8*, *ND2*, *ND4*) with TAG, and the remainder with TAA. The rRNA genes were 824 bp (*rrnS*) and 1,314 bp (*rrnL*). Twenty-two tRNA genes were identified, ranging in size from 62 bp for *trnL2(TAA)* to 71 bp for *trnY(GTA)*. The 844 bp control region (D-loop), positioned between *rrnS* and *trnI(GAT)*, exhibited a high AT content of 77.1%, exceeding that of the complete genome.

**Figure 3.**
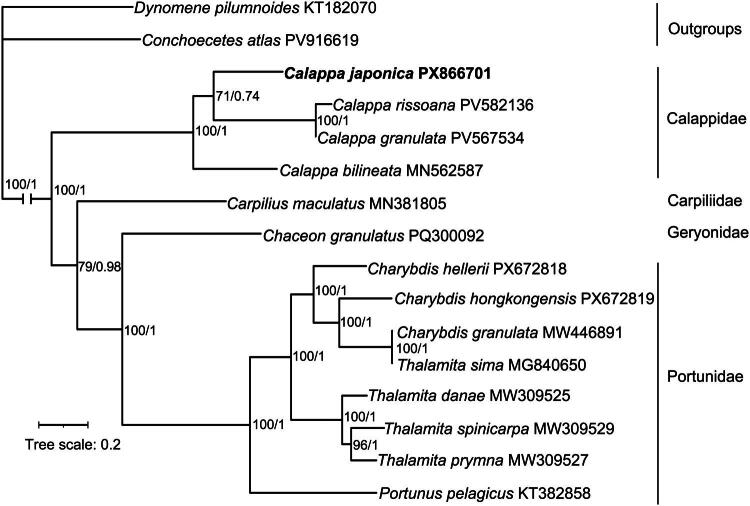
Maximum likelihood (ML) phylogenetic tree was constructed using maximum likelihood based on 13 protein-coding genes and two rRNA genes. Bootstrap support (BS) and bayesian posterior probabilities (PP) values were shown on the nodes. Tree scale represents substitutions per site. The long branch of outgroups was truncated. All species are labeled with their scientific names, GenBank accession numbers and family name on the right side. The position of *calappa japonica* is highlighted in bold.

The ML and BI phylogenetic analyses produced congruent topologies (Figure 3). *C. japonica* formed a well-supported clade with *C. granulata* and *C. rissoana* (BS = 71, PP = 0.74), and together with *C. bilineata* constituted the monophyletic family Calappidae (BS = 100, PP = 1). Calappidae was recovered as sister to a large clade comprising Geryonidae, Portunidae and Carpiliidae (BS = 100, PP = 1). The two outgroup taxa formed a well-supported monophyletic clade in both analyses (BS = 100, PP = 1).

## 4. Discussion and conclusion

This study reports the complete mitochondrial genome of *C. japonica*, adding the fourth mitogenomic resource for the family Calappidae and providing a genomic baseline for future comparative studies. The genome exhibits a typical brachyuran gene order and a strong AT bias (69.3%), with its overall structure and compositional characteristics highly similar to its congeners *C. bilineata*, *C. granulata* and *C. rissoana* (Lu et al. 2020; Tanduo et al. 2026). Phylogenetic analysis confirmed the monophyly of Calappidae with high support and revealed that *C. japonica* is more closely related to *C. granulata* and *C. rissoana* than to *C. bilineata*. At the family level, Calappidae was recovered as sister to a large clade comprising Geryonidae, Portunidae, and Carpiliidae.

Given that only three of the 88 described Calappidae species (≈4.5%) currently have available mitogenomes, further sequencing efforts are needed to construct a more comprehensive phylogenetic framework and to better understand the evolutionary history of this group.

## Supplementary Material

Supplementary_Table.docx

Figure S1.png

## Data Availability

The genome sequence data that support the findings of this study are openly available in GenBank of NCBI at https://www.ncbi.nlm.nih.gov under the accession no. PX866701. The associated BioProject, SRA, and Bio-Sample numbers are PRJNA1403095, SRR36840905, and SAMN54620113 respectively.
