# Supplementary material for: The complete mitogenome of Calappa japonica ortmann, 1892 (Decapoda: Calappidae) from the East China Sea
Source: Mitochondrial DNA B Resour. 2026 Apr 10;11(5):609–13. doi: 10.1080/23802359.2026.2657107 (PMC13072686; doi:10.1080/23802359.2026.2657107)
Supplement: Supplementary_Table.docx [file TMDN_A_2657107_SM5284.docx]

Supplementary material for:

The complete mitogenome of *Calappa japonica* Ortmann, 1892 (Decapoda: Calappidae) from the East China Sea

| Partition | Content | Best‑fit Model | Partition | Content | Best‑fit Model |
| --- | --- | --- | --- | --- | --- |
| 1 | ATP6_codon1 | *TIM2+F+R2* | 21 | ND1_codon3 | *HKY+F+R2* |
| 2 | ATP6_codon2 | *TVM+F+I* | 22 | ND2_codon1 | *GTR+F+R2* |
| 3 | ATP6_codon3 | *TN+F+I+G4* | 23 | ND2_codon2 | *TVM+F+G4* |
| 4 | ATP8_codon1 / ND6_codon1 | *TIM3+F+I+G4* | 24 | ND2_codon3 | *TN+F+G4* |
| 5 | ATP8_codon2 | *TPM3u+F+I* | 25 | ND3_codon1 | *TN+F+I+G4* |
| 6 | ATP8_codon3 | *K3Pu+F+I* | 26 | ND3_codon2 | *TIM3+F+I* |
| 7 | COX1_codon1 | *SYM+I+G4* | 27 | ND3_codon3 | *TN+F+R2* |
| 8 | COX1_codon2 | *TVM+F+I* | 28 | ND4L_codon1 | *TN+F+G4* |
| 9 | COX1_codon3 | *TIM+F+I+R3* | 29 | ND4L_codon2 | *TVM+F+G4* |
| 10 | COX2_codon1 | *GTR+F+G4* | 30 | ND4L_codon3 | *TN+F+I+G4* |
| 11 | COX2_codon2 | *TPM3u+F+I* | 31 | ND4_codon1 | *TIM+F+I+G4* |
| 12 | COX2_codon3 | *HKY+F+R3* | 32 | ND4_codon2 | *GTR+F+I+G4* |
| 13 | COX3_codon1 | *GTR+F+G4* | 33 | ND4_codon3 | *TN+F+R3* |
| 14 | COX3_codon2 | *K3Pu+F+I* | 34 | ND5_codon1 | *TIM+F+I+G4* |
| 15 | COX3_codon3 | *TIM3+F+R2* | 35 | ND5_codon2 | *GTR+F+I+R2* |
| 16 | CYTB_codon1 | *TIM2+F+I+G4* | 36 | ND5_codon3 | *TN+F+I+R2* |
| 17 | CYTB_codon2 | *GTR+F+G4* | 37 | ND6_codon2 | *TIM3+F+G4* |
| 18 | CYTB_codon3 | *TPM3u+F+I+R2* | 38 | ND6_codon3 | *HKY+F+R2* |
| 19 | ND1_codon1 | *GTR+F+I+G4* | 39 | rrnL | *GTR+F+R3* |
| 20 | ND1_codon2 | *TVM+F+R2* | 40 | rrnS | *TPM3u+F+I+G4* |

**Supplementary Table S1.** Partition schemes and best-fit substitution models used in Maximum Likelihood (ML) phylogenetic analysis.

**Note:** Model selection was performed using ModelFinder v3.2 in IQ‑TREE v2.2.0. “+F” indicates empirical base frequencies; “+I” indicates a proportion of invariable sites; “+G4” indicates a discrete gamma distribution with four rate categories; “+R2” and “+R3” indicate the free‑rate model with two or three rate categories, respectively.

| Partition | Substitution Model | Rate Heterogeneity | Model Selected |
| --- | --- | --- | --- |
| 1 | 6 (GTR) | Invgamma | *GTR + I + Γ* |
| 2 | 6 (GTR) | Invgamma | *GTR + I + Γ* |
| 3 | 6 (GTR) | Invgamma | *GTR + I + Γ* |
| 4 | 6 (GTR) | Invgamma | *GTR + I + Γ* |
| 5 | 6 (GTR) | Propinv | *GTR + I* |
| 6 | 6 (GTR) | Gamma | *GTR + Γ* |
| 7 | 6 (GTR) | Propinv | *GTR + I* |
| 8 | 6 (GTR) | Invgamma | *GTR + I + Γ* |
| 9 | 6 (GTR) | Gamma | *GTR + Γ* |
| 10 | 6 (GTR) | Invgamma | *GTR + I + Γ* |
| 11 | 6 (GTR) | Invgamma | *GTR + I + Γ* |
| 12 | 2 (HKY) | Gamma | *HKY + Γ* |
| 13 | 6 (GTR) | Invgamma | *GTR + I + Γ* |
| 14 | 6 (GTR) | Gamma | *GTR + Γ* |
| 15 | 6 (GTR) | Gamma | *GTR + Γ* |
| 16 | 6 (GTR) | Gamma | *GTR + Γ* |
| 17 | 2 (HKY) | Propinv | *HKY + I* |
| 18 | 6 (GTR) | Invgamma | *GTR + I + Γ* |
| 19 | 6 (GTR) | Invgamma | *GTR + I + Γ* |
| 20 | 6 (GTR) | Invgamma | *GTR + I + Γ* |
| 21 | 6 (GTR) | Gamma | *GTR + Γ* |
| 22 | 6 (GTR) | Invgamma | *GTR + I + Γ* |

Supplementary material for:

The complete mitogenome of *Calappa japonica* Ortmann, 1892 (Decapoda: Calappidae) from the East China Sea

**Supplementary Table S2.** Partition schemes and substitution models used in Bayesian Inference (BI) phylogenetic analysis.

**Note:** Model selection was performed using PartitionFinder 2 in MrBayes v3.2. Models were chosen from GTR and HKY families, with rate heterogeneity modeled via gamma (G) or invariable sites (I). “GTR” and “HKY” denote the general time-reversible and Hasegawa – Kishino - Yano substitution models, respectively. “I” represents the proportion of invariable sites, and “Γ” represents the discrete gamma distribution.
